# *Angiostrongylus cantonensis* and *A*. *malaysiensis* Broadly Overlap in Thailand, Lao PDR, Cambodia and Myanmar: A Molecular Survey of Larvae in Land Snails

**DOI:** 10.1371/journal.pone.0161128

**Published:** 2016-08-11

**Authors:** Rutchanee Rodpai, Pewpan M. Intapan, Tongjit Thanchomnang, Oranuch Sanpool, Lakkhana Sadaow, Sakhone Laymanivong, Win Papa Aung, Issarapong Phosuk, Porntip Laummaunwai, Wanchai Maleewong

**Affiliations:** 1 Department of Parasitology, Faculty of Medicine, Khon Kaen University, Khon Kaen, Thailand; 2 Research and Diagnostic Center for Emerging Infectious Diseases, Faculty of Medicine, Khon Kaen University, Khon Kaen, Thailand; 3 Faculty of Medicine, Mahasarakham University, Mahasarakham, Thailand; 4 Laboratory Unit, Centre of Malariology, Parasitology and Entomology, Ministry of Health, Vientiane, Lao People's Democratic Republic; 5 Department of Microbiology, University of Medicine 2, Yangon, Myanmar; Australian Museum, AUSTRALIA

## Abstract

*Angiostrongylus cantonensis* is a zoonotic nematode parasite causing human eosinophilic meningitis (or meningoencephalitis) worldwide. A closely related species, *Angiostrongylus malaysiensis*, might also be a human pathogen. Larvae were obtained from land snails in Lao PDR, Cambodia, Myanmar and Thailand. We sequenced two nuclear gene regions (nuclear ribosomal ITS2 and SSU rRNA) and a portion of one mitochondrial gene (COI) from these larvae. *Angiostrongylus cantonensis* and *A*. *malaysiensis* were identified. This is the first report of the molecular identification of the two *Angiostrongylus* species in Lao PDR, Cambodia and Myanmar. The regional distributions of the two species broadly overlap. Phylogenetic relationships were inferred including data from *Angiostrongylus* species deposited in public databases. All the gene regions we sequenced have potential value in distinguishing between species of *Angiostrongylus*. The COI gene exhibited the greatest intraspecific variation in the study region (five haplotypes in *A*. *cantonensis* and four in *A*. *malaysiensis*) and might be suitable for more detailed phylogeographic studies.

## Introduction

The genus *Angiostrongylus* contains nematodes parasitic in rodents and carnivores, in which they are found in the mesenteric or pulmonary arteries and lungs [1]. *Angiostrongylus cantonensis* is a primary cause of human eosinophilic meningitis or meningoencephalitis in many areas of the world: more than 2800 cases have been documented worldwide [2–6]. Humans are incidental hosts who become infected by eating infective larvae in snails, slugs, paratenic hosts or contaminated vegetables [4,7]. Additional species have been reported. *Angiostrongylus costaricensis* causes abdominal angiostrongyliasis in Latin American countries [8]. *Angiostrongylus mackerrasae* and *Angiostrongylus malaysiensis* may also be pathogenic in humans [9]. Identification of *Angiostrongylus* species based on morphological characters is difficult due to vague and similar descriptions of size and body shapes among different species [1,10,11]. Molecular methods, based on PCR-direct sequencing of the nuclear small subunit ribosomal RNA (SSU rRNA) gene, now provide a useful approach for identification of *Angiostrongylus* species [12]. The mitochondrial cytochrome c subunit I (COI) gene and the nuclear ribosomal internal transcribed spacer 2 (ITS2) region have also been used for constructing phylogenetic trees to differentiate geographical isolates of *A*. *cantonensis*, *A*. *malaysiensis*, *A*. *costaricensis* and *Angiostrongylus vasorum* [13–17]. Despite several reports of angiostrongyliasis in Thailand, confirmed using molecular methods [2], there have been no reports on molecular identification of *Angiostrongylus* species in Lao PDR, Myanmar and Cambodia, countries adjacent to Thailand in the Greater Mekong subregion. Therefore, in the present study, we characterized the DNA regions of SSU rRNA, ITS2 and COI of *A*. *cantonensis* and *A*. *malaysiensis* from these countries and their phylogenetic relationships with parasites recovered from Thailand elsewhere. Intra- and interspecific variation are discussed.

## Materials and Methods

### *Angiostrongylus* worms

**Fig 1:**
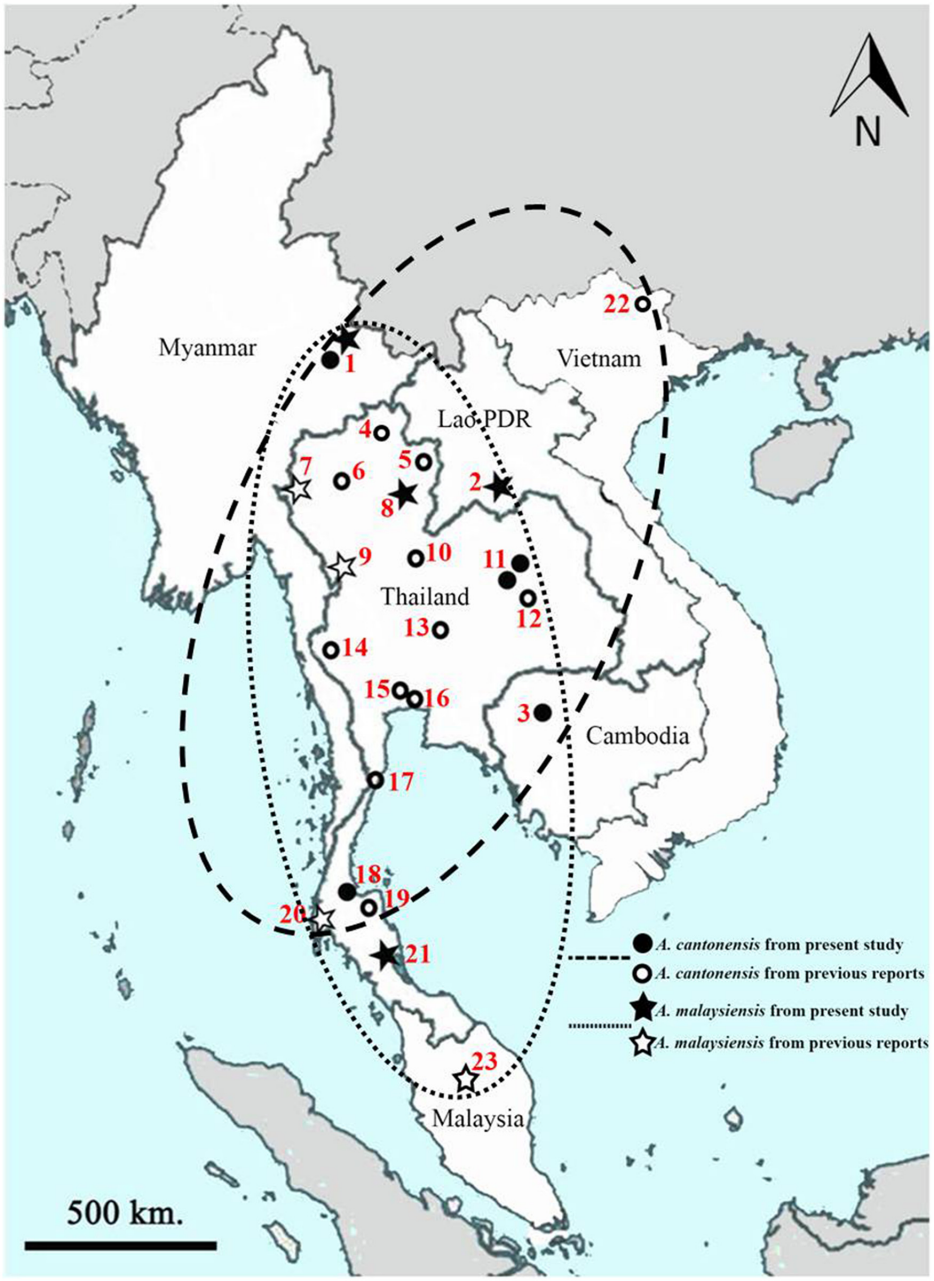
Map of sampling locations of *Angiostrongylus cantonensis* and *Angiostrongylus malaysiensis* showing their overlapping distributions in six countries. Stars indicate *A*. *malaysiensis*; circles indicate *A*. *cantonensis*; the dotted line shows inferred range of *A*. *malaysiensis*; the dashed line shows range of *A*. *cantonensis* in the region. 1, Mongla Township in Myanmar (present study); 2, Vientiane capital city in Lao PDR (present study); 3, Siem Reap Province of Cambodia (present study); 4–21, Provinces in Thailand (4, Chiang Rai (KP721449); 5, Nan (KC995246); 6, Chiang Mai (KP721447); 7, Mae Hong Sorn (KP732099); 8, Phrae (present study); 9, Tak (KP732097); 10, Phitsanulok (KP721445); 11, Khon Kaen (present study); 12, Maha-Sarakham (KC995223); 13, Lopburi (KC995212); 14, Kanchanaburi (KC995206); 15, Bangkok (KC995188); 16, Samut Prakan (KP721442); 17, Prachuap Khiri Khan (KC995262); 18, Surat Thani (present study); 19, Nakhon Si Thammarat (KC995260); 20, Phang Nga (KP732100); 21, Phattalung (present study)); 22, Cao Bang Province of Vietnam (De et al [18]); 23, Peninsular Malaysia (JN663729).

Third-stage *Angiostrongylus* larvae (L3) recovered from land snails in Thailand came from the Northeast, Khon Kaen Province, the North, Phrae Province, and the South, Surat Thani and Patthalung Provinces. The L3 larvae from Myanmar were recovered from *Achatina fulica* collected from Mongla Township, Shan State, eastern Myanmar. The L3 larvae from Laos were recovered from *Cryptozona siamensis* from Vientiane, central Lao PDR. The L3 larvae from Cambodia were recovered from *Ac*. *fulica* from Siem Reap Province, northwestern Cambodia (kindly provided by Dr. Chivorn Leang, Phnom Penh, Cambodia). All snails were bought from daily food markets in each of these countries. The collection localities and host species of *Angiostrongylus* samples are presented in Fig 1. An adult *A*. *cantonensis* of the Hawaiian strain (kindly provided by Professor Yukifumi Nawa, Research Affairs, Faculty of Medicine, Khon Kaen University, Khon Kaen, Thailand) was used as the reference sample from outside Asian countries. Information about samples is in Table 1. The parasite samples were kept in 75% ethanol at -70°C until used. These data do not report any studies with animals performed by any of the authors. We confirm that the process did not involve endangered or protected species.

**Table 1:** *Angiostrongylus* worms used in the present study and the mitochondrial COI haplotypes found.

Locality	Worm species	No specimens *	Source host	Date	GenBank no. of COI gene	COI Haplotypes
Mancha Khiri, Khon Kaen, Thailand	*A*. *cantonensis*	1	*Achatina fulica*	2013	KU532143	AC11
Mueang, Khon Kaen, Thailand	*A*. *cantonensis*	1	*Ac*. *fulica*	2010	KU532147	AC10
Surat Thani, Thailand	*A*. *cantonensis*	1	*Ac*. *fulica*	2014	KU532146	AC13
Siem Reap, Cambodia	*A*. *cantonensis*	10	*Ac*. *fulica*	2015	KU532148	AC12
Mongla, Shan State, Myanmar	*A*. *cantonensis*	7	*Ac*. *fulica*	2013	KU532145	AC2
Hawaii, United States	*A*. *cantonensis*	1	Experimentally maintained rat	NA	KU532144	AC5
Phatthalung, Thailand	*A*. *malaysiensis*	3	*Ac*. *fulica*	2014	KU532152	AM1
Phrae, Thailand	*A*. *malaysiensis*	2	*Cryptozona siamensis*	2014	KU532151	AM3
Vientiane, Lao PDR	*A*. *malaysiensis*	2	*C*. *siamensis*	2015	KU532153	AM3
Vientiane, Lao PDR	*A*. *malaysiensis*	8	*C*. *siamensis*	2015	KU532154	AM4
Mongla, Shan State, Myanmar	*A*. *malaysiensis*	1	*Ac*. *fulica*	2013	KU532149	AM3
Mongla, Shan State, Myanmar	*A*. *malaysiensis*	2	*Ac*. *fulica*	2013	KU532150	AM2

NA, not available.

*All specimens were third stage larvae, except for one adult (Hawaii isolate).

### DNA extraction, PCR amplification and DNA sequencing

Extraction of genomic DNA from each *Angiostrongylus* sample was done using a Genomic DNA Mini Kit (Macherey-Nagel GmbH & Co., Duren, Germany) according to the manufacturer’s instructions. Specific primers and regions amplified are listed in Table 2. All polymerase chain reactions (PCRs) were done using a GeneAmp PCR System 9700 (Applied Biosystems, Singapore). The reaction mixture was prepared in a total volume of 25 μL containing 2.5 μL of 10x high fidelity PCR buffer, 2.0 μL MgCl_2_ (25 mM), 0.5μL of dNTP mix (10 mM), 0.5 μL of each primer (10 μM), 0.125 U of Taq high-fidelity PCR system (Roche Applied Science, Mannheim, Germany), adjusted to 25 μL with deionized water and 5 μL of DNA extracted from an individual worm. For the SSU rRNA and the COI amplifications, the PCR conditions were initial denaturation at 94°C for 2 min, followed by 10 cycles of denaturation at 94°C for 1 min, annealing at 40°C for 1 min and extension at 68°C for 2 min, then 30 cycles of denaturation at 94°C for 1 min, annealing at 45°C for 2 min, extension at 68°C for 2 min and a final extension at 68°C for 7 min. For the ITS2 amplification, the PCR conditions were initial denaturation at 94°C for 5 min, followed by 35 cycles of denaturation at 94°C for 30 sec, annealing at 55°C for 30 sec, extension at 72°C for 30 sec and final extension at 72°C for 7 min. Amplified products were separated by electrophoresis on a 1.0% (w/v) agarose gel, stained with ethidium bromide, and visualized under ultraviolet light. DNA sequencing was conducted at First BASE Laboratories Sdn Bhd (Selangor, Malaysia) using the BigDye terminator v3.1 cycle-sequencing kit (ABI), and both strands were directly sequenced using the PCR primers as sequencing primers (Model 310 or 3100, Applied Biosystems).

**Table 2:** The specific primers used in the present study.

Gene regions	Primers	Approximate amplicon size
SSU rRNA	Angi18S-1_forward 5’-AAAGTTAAGCCATGCATG-3’ Angi18S-2_reverse 5’-CATTCTTGGCAAATGCTTTCG-3’ [17]	885 bp
COI	AngiCOI_forward 5’-TTTTTTGGGCATCCTGAGGTTTAT-3’ AngiCOI_reverse 5’-CGAGGATAACCATGTAAACCAGC-3’ (newly designed primers)	605 bp
ITS2	AngiITS2_ forward 5' ACATCTGGTTCAGGGTTGTT 3' AngiITS2_ reverse 5' AGCATACAAGCACATGATCAC 3' (newly designed primers)	395 bp

### Sequence alignment and Phylogenetic analysis

The sequences were aligned and compared with those of *Angiostrongylus* species deposited in the GenBank database using the multiple sequence alignment program ClustalW available within BioEdit (http://www.mbio.ncsu.edu/bioedit/bioedit.html) [19]. Phylogenetic trees were constructed using the maximum-likelihood (ML), maximum-parsimony (MP) and neighbour-joining (NJ) methods implemented in MEGA6 [20]. The substitution model for each dataset was chosen using the Bayesian Information Criterion (BIC) in MEGA6 software: the lowest BIC score is considered to best describe the substitution pattern [20, 21]. Models used were the Kimura two-parameter (K2) model (SSU rRNA alignment), Tamura three-parameter (T92) model (ITS2 alignment) and the General Time Reversible model allowing for a proportion of invariable sites (GTR+I) (COI alignment). Bootstrap percentages were computed using 1000 pseudoreplications. Bayesian analyses were performed using MrBayes v3.2. [22]. MrBayes implements a more limited number of substitution models than does MEGA6. The available model with the closest BIC score was therefore chosen. For the SSU rRNA alignment, this was the Hasegawa-Kishino-Yano model (HKY). The SSU analysis was run for three million generations (2 runs, each of four chains), by which time the standard deviation of split frequencies had fallen below 0.01, and sampled every 1000 generations. Examination of the output (using the “sump” command) indicated that the potential scale reduction factor was 1 for relevant parameters and that stationarity had been approached after 20% of generations. The first 20% of trees were therefore discarded as burnin (following recommendations in the MrBayes manual). For ITS2, the best model was the HKY model allowing for a proportion of invariable sites (HKY+I). Following the approach outlined above for the SSU alignment, the analysis was run for two million generations and the first 25% of trees discarded as burnin. For COI, given that this is a protein-coding gene and that each codon position might evolve in a different way, a different approach was used. Partition Finder v1 [23] indicated that the same substitution model should be applied to each codon position and that, for implementation in MrBayes, this model was the GTR+I. The analysis was run for five million generations and sampled every 1000 generations. The first 20% of trees were discarded as burnin. In all Bayesian analyses, consensus trees were generated using the command “contype = allcompat”, which adds all compatible groups to a 50% majority-rule tree. The COI haplotypes were numbered following Monte et al [16].

## Results

A total of 39 *Angiostrongylus* specimens were used for PCR amplification and DNA sequencing (Table 1). A single representative of each unique nucleotide sequence from each collection site was retained for phylogenetic analysis (Fig 1). Ten new partial SSU rRNA and ITS2 sequences and 12 new partial COI sequences were used for phylogenetic analyses (GenBank accession numbers KU528678-KU528687, KU528688-KU528697 and KU532143-KU532154, respectively). The distributions of *A*. *cantonensis* and *A*. *malaysiensis* were found to overlap broadly in the region.

### Small subunit ribosomal RNA gene

**Fig 2:**
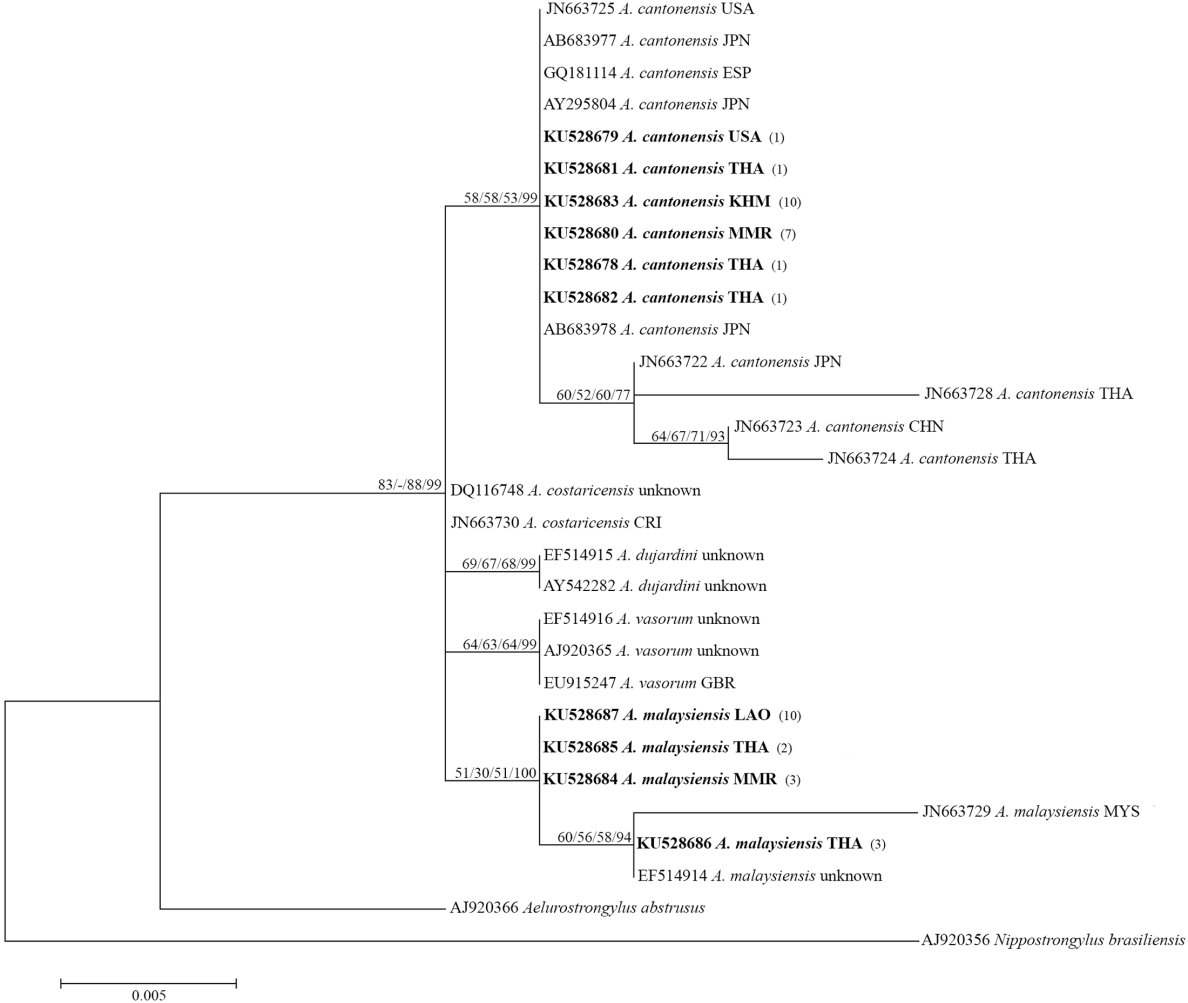
Maximum-likelihood phylogenetic tree of five species of *Angiostrongylus* (*A*. *cantonensis*, *A*. *malaysiensis*, *A*. *dujardini*, *A*. *costaricensis* and *A*. *vasorum*), *Aelurostrogylus abstrusus* and *Nippostronglus braziliensis* (outgroups) based on partial SSU rRNA sequences. Support values (ML bootstrap/MP bootstrap/NJ bootstrap/Bayesian posterior probabilities) are shown above the branches. A dash (-) instead of a numerical support value indicates that a particular grouping was not found by that method of analysis. Bold letters indicate sequences obtained in the present study. Numbers in parentheses following each newly obtained sequence indicate the number of larvae possessing that sequence.

The two major species were clearly distinguished according to SSU rRNA sequences, as shown in Fig 2. Bootstrap/Bayesian posterior probability values of 58/58/53/99% and 51/30/51/100% supported *A*. *cantonensis* and *A*. *malaysiensis*, respectively. All new *A*. *cantonensis* SSU rRNA sequences were almost identical, regardless of geographical origin. However, there was slight variation among *A*. *malaysiensis* sequences. Based on analysis of 786 nucleotides between *A*. *cantonensis* and *A*. *malaysiensis*, a total of 8 variable sites were found (Table 3).

**Table 3:** Variable nucleotide positions within the SSU rRNA gene of *A*. *cantonensis* and *A*. *malaysiensis*.

Nucleotide positions*	29	181	227	480	656	662	665	689
*A*. *cantonensis* (AY295804)	T	T	C	C	T	G	A	A
***A*.*cantonensis* THA (KU528678)**	T	T	C	C	T	G	A	A
***A*.*cantonensis* THA (KU528682)**	T	T	C	C	T	G	A	A
***A*.*cantonensis* THA (KU528681)**	T	T	C	C	T	G	A	A
***A*.*cantonensis* KHM (KU528683)**	T	T	C	C	T	G	A	G
***A*.*cantonensis* MMR (KU528680)**	T	T	C	C	T	G	A	G
***A*.*cantonensis* USA (KU528679)**	T	T	C	C	T	G	A	A
*A*. *malaysiensis* (EF514914)	C	C	T	A	C	A	G	G
***A*.*malaysiensis* THA (KU528686)**	C	C	T	A	C	A	G	G
***A*.*malaysiensis* THA (KU528685)**	C	T	T	A	C	G	G	G
***A*.*malaysiensis* LAO (KU528687)**	C	T	T	A	C	G	G	G
***A*.*malaysiensis* MMR (KU528684)**	C	T	T	A	C	G	G	G

THA, Thailand; KHM, Cambodia; MMR, Myanmar; USA, United States of America; LAO, Lao PDR.

* Based on nucleotide positions in the *A*. *cantonensis* SSU rRNA sequence (AY295804). Bold letters indicated the present results.

### Ribosomal DNA internal transcribed spacer 2 gene

**Fig 3:**
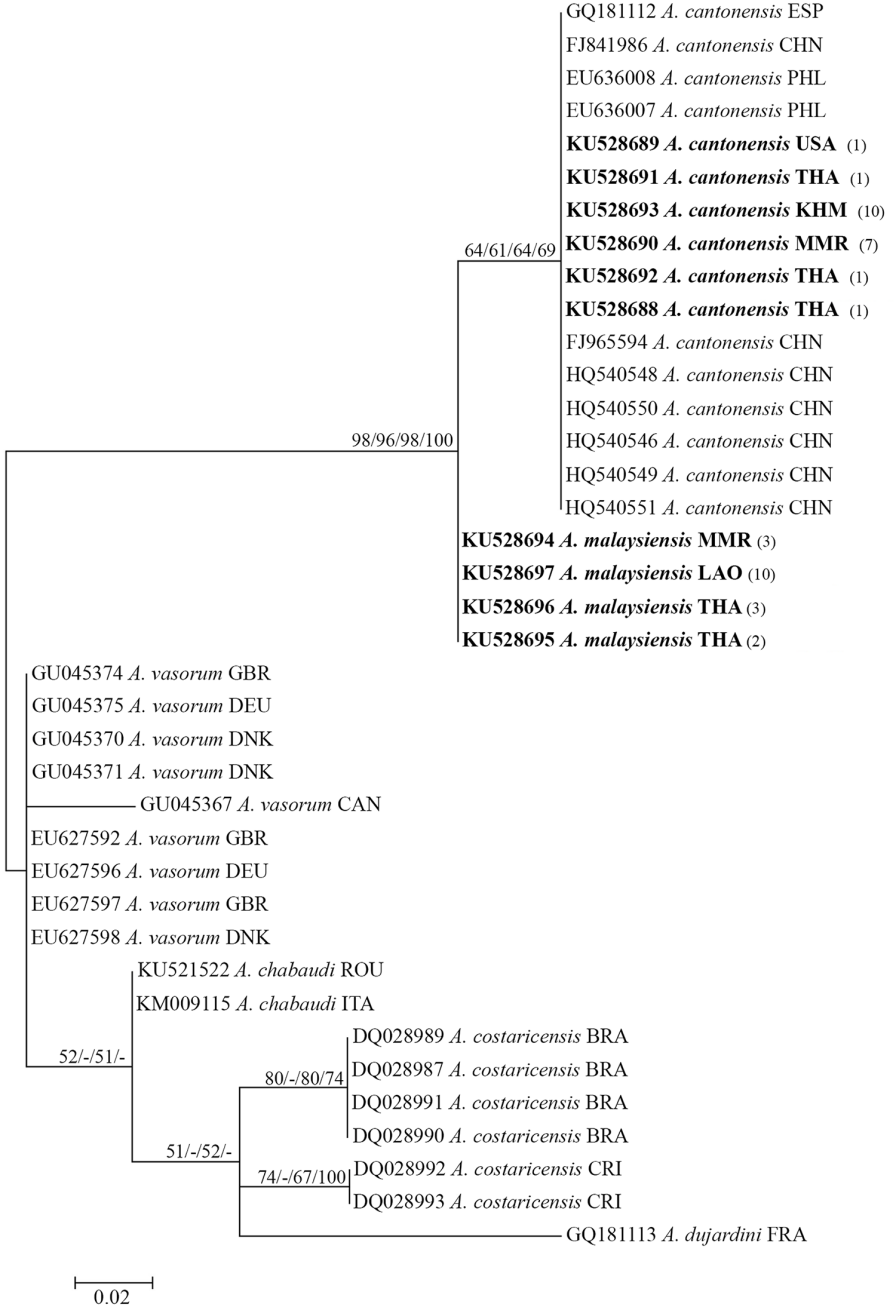
Maximum-likelihood phylogenetic tree of *Angiostrongylus cantonensis*, *A*. *malaysiensis*, *A*. *vasorum*, *A*. *chabaudi*, *A*. *costaricensis* and *A*. *dujardini* based on the partial ITS2 sequences. Support values (ML bootstrap/MP bootstrap/NJ bootstrap/Bayesian posterior probabilities) are shown above the branches. A dash (-) instead of a numerical support value indicates that a particular grouping was not found by that method of analysis. Bold letters indicate sequences obtained in the present study. Numbers in parentheses following each newly obtained sequence indicate the number of larvae possessing that sequence.

The phylogenetic tree based on the *Angiostrongylus* ITS2 sequences is shown in Fig 3. No outgroup was specified because of the difficulty in aligning species from other genera or families–this is a midpoint-rooted tree. Even within the genus *Angiostrongylus*, each species exhibited many unique repeats and indels. All *A*. *cantonensis* sequences fell into a well-supported group (bootstrap/Bayesian posterior probability values 64/61/64/69%). All *A*. *malaysiensis* sequences from Lao PDR, Myanmar and two provinces in Thailand (Phrae and Phatthalung) were identical, forming a group with 100% Bayesian posterior probability (Bayesian Inference, figure not shown).

### Mitochondrial cytochrome *c* oxidase subunit I gene

**Fig 4:**
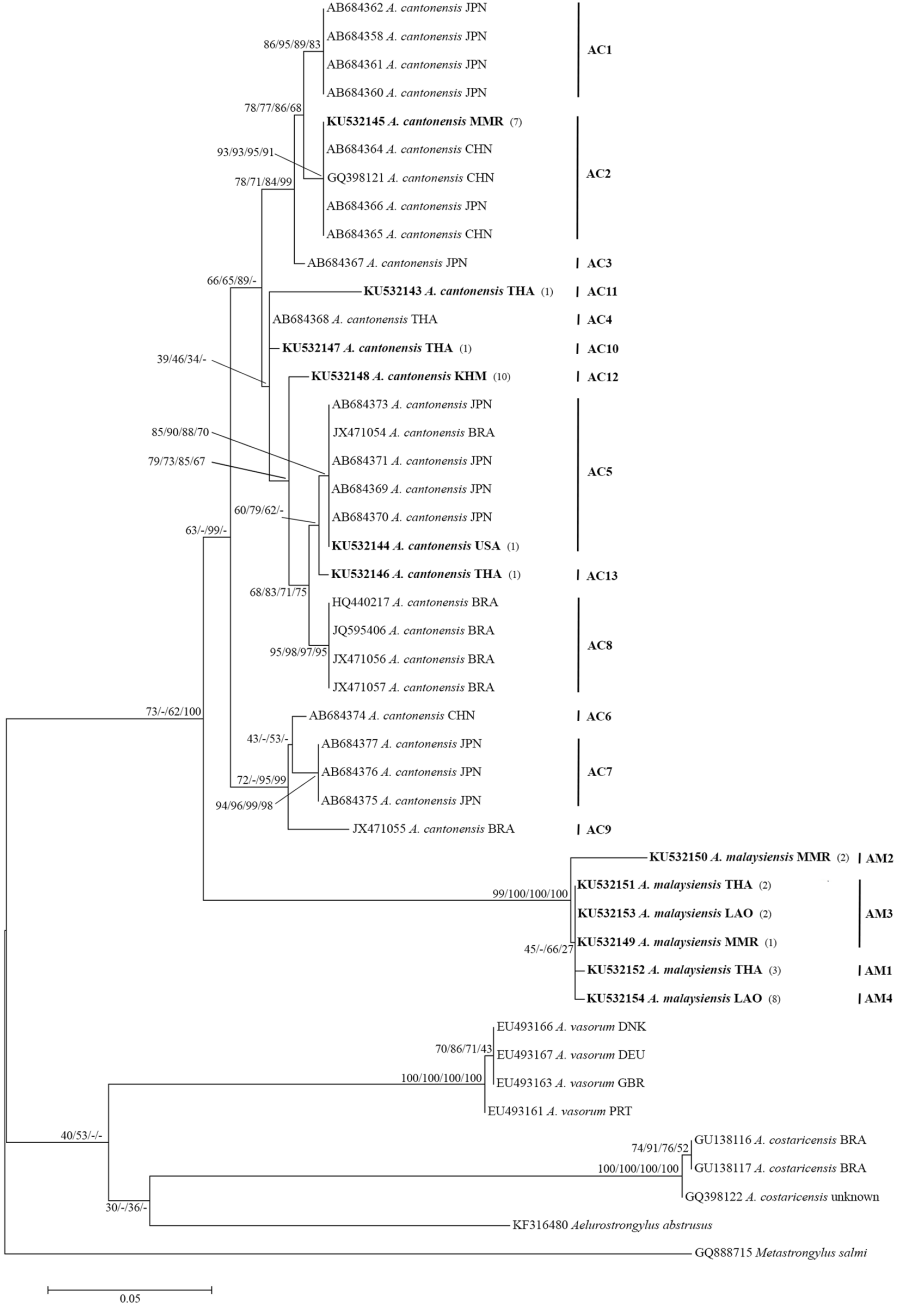
Maximum-likelihood phylogenetic tree of *Angiostrongylus cantonensis*, *A*. *malaysiensis*, *A*. *vasorum*, *A*. *costaricensis*, *Aelurostrogylus abstrusus* and *Metastrongylus salmi* (the last two as outgroups) based on partial COI sequences. Support values (ML bootstrap/MP bootstrap/NJ bootstrap/Bayesian posterior probabilities) are shown above the branches. A dash (-) instead of a numerical support value indicates that a particular grouping was not found by that method of analysis. Bold letters indicate sequences obtained in the present study. AC1-AC13 are the 13 distinct haplotypes of *A*. *cantonensis*; AM1-AM4 are the 4 distinct haplotypes of *A*. *malaysiensis*. Numbers in parentheses following each newly obtained sequence indicate the number of larvae possessing that sequence.

In the phylogenetic analysis based on the COI sequences, all *A*. *cantonensis* formed a monophyletic cluster with support values of 63/-/99/-%. The *A*. *cantonensis* sequences fell into 13 distinct haplotypes (four of them, AC10-AC13, not previously observed–Table 1) when all previously published data were included (Fig 4). The *A*. *malaysiensis* sequences formed a second monophyletic cluster with support values of 99/100/100/100%. This cluster contained four distinct haplotypes (AM1-AM4). Interspecific distances between *A*. *cantonensis* and *A*. *malaysiensis* sequences ranged from 9% to 13%. Intraspecific distances within *A*. *cantonensis* ranged from <1% to 4%, and among *A*. *malaysiensis* from <1 to 2% (Table 4).

**Table 4:** Pairwise *p*-distances between COI sequences from *A*. *cantonensis* and *A*. *malaysiensis* samples.

	1	2	3	4	5	6	7	8	9	10	11	12	13	14
1. GQ398121 *A*.*cantonensis* CHN	-													
2. AB684368 *A*.*cantonensis* THA	0.02	-												
3. AB684370 *A*.*cantonensis* JPN	0.03	0.01	-											
4. **KU532143 *A*.*cantonensis* THA**	0.03	0.02	0.03	-										
5. **KU532147 *A*.*cantonensis* THA**	0.02	0.00	0.02	0.03	-									
6. **KU532145 *A*.*cantonensis* MMR**	0.00	0.02	0.03	0.03	0.02	-								
7. **KU532146 *A*.*cantonensis* THA**	0.04	0.02	0.01	0.04	0.02	0.04	-							
8. **KU532144 *A*.*cantonensis* USA**	0.03	0.01	0.00	0.03	0.02	0.03	0.01	-						
9. **KU532148 *A*.*cantonensis* KHM**	0.03	0.01	0.01	0.03	0.01	0.03	0.02	0.01	-					
10. **KU532149 *A*.*malaysiensis* MMR**	0.10	0.10	0.11	0.11	0.10	0.10	0.11	0.11	0.11	-				
11. **KU532153 *A*.*malaysiensis* LAO**	0.10	0.10	0.11	0.11	0.10	0.10	0.11	0.11	0.11	0.00	-			
12. **KU532154 *A*.*malaysiensis* LAO**	0.10	0.09	0.10	0.10	0.10	0.10	0.11	0.10	0.10	0.00	0.00	-		
13. **KU532151 *A*.*malaysiensis* THA**	0.10	0.10	0.11	0.11	0.10	0.10	0.11	0.11	0.11	0.00	0.00	0.00	-	
14. **KU532152 *A*.*malaysiensis* THA**	0.10	0.10	0.11	0.11	0.11	0.10	0.11	0.11	0.11	0.00	0.00	0.01	0.01	-
15. **KU532150 *A*.*malaysiensis* MMR**	0.12	0.12	0.13	0.13	0.12	0.12	0.13	0.13	0.13	0.02	0.02	0.02	0.02	0.02

CHN, China; THA, Thailand; JPN, Japan; MMR, Myanmar; USA, United States of America; KHM, Cambodia; LAO, Lao PDR.

Bold letters indicated the present results.

## Discussion

This study investigated the distribution and genetic relationships (based on SSU rRNA, ITS2 and COI nucleotide sequences) of *A*. *cantonensis* and *A*. *malaysiensis* specimens originating from land snails from different locations in Lao PDR, Cambodia, Myanmar and Thailand. To our knowledge, this is the first report of *A*. *cantonensis* in Cambodia and Myanmar, and also the first report of *A*. *malaysiensis* in Lao PDR and Myanmar. Distributions of these two species in the four countries broadly overlap (Fig 1). A recent molecular study in Thailand using mitochondrial cytochrome b (Cytb) nucleotide sequences reported *A*. *cantonensis* in two central provinces (Samut Prakan and Bangkok), two northern provinces (Phitsanulok and Chiang Mai), a northeastern province (Khon Kaen) and in southern province (Surat Thani) [24]. The same study reported *A*. *malaysiensis* from the north (Tak and Mae Hong Sorn) and south (Phang Nga) of Thailand. Our study found *A*. *cantonensis* in northeastern Thailand (Khon Kaen Province) and in the south (Surat Thani Province). We found *A*. *malaysiensis* in Phrae Province (northern Thailand) and Phatthalung Province (in the south). Interestingly, we also found *A*. *cantonensis* in eastern Myanmar and northwestern Cambodia and *A*. *malaysiensis* in central Lao PDR and eastern Myanmar.

There is little intraspecific variation of the SSU rRNA nucleotide sequences in *A*. *cantonensis* and *A*. *malaysiensis* (Table 3). Consequently, the 786-bp fragment of the SSU rRNA gene is a suitable marker to identify *A*. *cantonensis* and distinguish it from other *Angiostrongylus* species [12,17].

ITS2 sequences revealed distinct differences among *A*. *cantonensis*, *A*. *malaysiensis*, *A*. *vasorum*, *A*. *chabaudi* and *A*. *costaricensis* as reported previously [14,15], but minimal or no differences within each species. This conserved region could therefore be a useful genetic marker for the specific identification and genetic characterization of *Angiostrongylus* species.

The COI gene is one of the most commonly used sources of sequence data for studying geographical populations of *Angiostrongylus* species [13,14,16,17]. The present study supports previous findings, based on COI and Cytb sequences, that *A*. *cantonensis* is more closely related to *A*. *malaysiensis* than to *A*. *costaricensis* and *A*. *vasorum* [13,16,17,24]. Within *A*. *cantonensis*, we identified 13 haplotypes, an increase on the 9 haplotypes reported by Monte et al. [16]. Two haplotypes (AC2 and AC5) (Fig 4) have previously been reported from *A*. *cantonensis* from Brazil and Asia [16]. One sample (n = 7 larvae) from Myanmar exhibited the AC2 haplotype. In addition, we found four new haplotypes (AC10-AC13) from Thailand and Cambodia (Fig 4). In *A*. *malaysiensis*, one haplotype (AM3) occurred in worms from Laos, Myanmar and Thailand. A separate, distinct haplotype was also found in some specimens from each of those countries. The analysis also indicates a *p*-distance between *A*. *cantonensis* and *A*. *malaysiensis* ranging from 9% to 13% (Table 4), similar to previously reported values [13]. Pair-wise intraspecific distances among *A*. *cantonensis* haplotypes ranged from <1% to 4%, and among *A*. *malaysiensis* from <1% to 2% (Table 4). This suggests that COI sequences might be useful for differentiation of geographical isolates [16].

## Conclusions

In summary, the present study has reported for the first time the phylogenetic relationships of *A*. *cantonensis* and *A*. *malaysiensis* in parts of the Greater Mekong subregion, based on sequences from three gene regions. The two species have broadly overlapping distributions in Lao PDR, Cambodia, Myanmar and Thailand. The techniques employed in this study can clearly be used in a molecular approach to identify *Angiostrongylus* species in the future. This provides a reliable alternative to morphological identification of parasite samples, especially in cases where morphological features are obscure in larval stages.
